# Computational Analysis on Antioxidant Activity of Four Characteristic Structural Units from Persimmon Tannin

**DOI:** 10.3390/ma16010320

**Published:** 2022-12-29

**Authors:** Zhongmin Wang, Zhigao Liu, Chenxi Wu, Songlin Liu, Dianhui Wang, Chaohao Hu, Tao Chen, Zhaojin Ran, Weijiang Gan, Guiyin Li

**Affiliations:** 1 Guangxi Academy of Sciences, Nanning 530007, China; 2 School of Materials Science and Engineering, Guilin University of Electronic Technology, Guilin 541004, China; 3 College of Chemistry, Guangdong University of Petrochemical Technology, Maoming 525000, China

**Keywords:** persimmon tannin, characteristic structural unit, antioxidant activity, density functional theory

## Abstract

Antioxidants are molecules that can prevent the harmful effects of oxygen, help capture and neutralize free radicals, and thus eliminate the damage of free radicals to the human body. Persimmon tannin (PT) has excellent antioxidant activity, which is closely related to its molecular structure. We report here a comparative study of four characteristic structural units from PT (epicatechin gallate (ECG), epigallocatechin gallate (EGCG), A−type linked ECG dimer (A−ECG dimer), A−type linked EGCG dimer (A−EGCG dimer)) to explore the structure–activity relationship by using the density functional theory. Based on the antioxidation mechanism of hydrogen atom transfer, the most favorable active site for each molecule exerts antioxidant activity is determined. The structural parameters, molecular electrostatic potential, and frontier molecular orbital indicate that the key active sites are located on the phenolic hydroxyl group of the B ring for ECG and EGCG monomers, and the key active sites of the two dimers are located on the phenolic hydroxyl groups of the A and D’ rings. The natural bond orbital and bond dissociation energy of the phenolic hydroxyl hydrogen atom show that the C_11_−OH in the ECG monomer and the C_12_−OH in the EGCG monomer are the most preferential sites, respectively. The most active site of the two A−linked dimers is likely located on the D’ ring C_20′_ phenolic hydroxyl group. Based on computational analysis of quantum chemical parameters, the A−ECG dimer is a more potent antioxidant than the A−EGCG dimer, ECG, and EGCG. This computational analysis provides the structure–activity relationship of the four characteristic units which will contribute to the development of the application of PT antioxidants in the future.

## 1. Introduction

Antioxidation is a process in which the antioxidant molecule fights against excess free radicals in the human body. Studies have shown that excess oxygen radicals in the body can cause damage to proteins, lipids, and DNA, which eventually bring aging and death to the organism [1,2]. Persimmon tannin (PT) is a unique flavonoid polyphenol compound in persimmons [3]. The repeating units of PT contain multiple active groups (ortho-phenolic hydroxyl groups), which make persimmon tannin perform well in anti-oxidation, deodorization, adsorption of metal ions, and protein binding [4,5,6]. Therefore, the antioxidant properties of PT have been explored by many researchers. Shin et al. [7] investigated the antioxidant properties of tannins in different types of persimmons by measuring DPPH radical scavenging activity, ABTS radical scavenging activity, etc. The results showed the presence of different antioxidant activities of soluble and insoluble PT depending on the type of persimmon. Chang et al. [8] analyzed several kinds of persimmon leaves harvested in different periods and verified that the harvest period and leaf types are important factors affecting the composition of phenolic compounds in persimmon leaves. However, experimental methods can show that a compound exhibits or does not exhibit antioxidant activity and enable us to compare the anti-oxidative potential of a set of molecules but are unable to show which parts or sites in molecules are responsible for the antioxidant action. Therefore, it is necessary to conduct theoretical research on the relationship between the antioxidant capacity and the structure and physical and chemical properties of compounds with antioxidant activity.

Density functional theory (DFT) is the most important computational method and has been widely used to analyze the antioxidant activity and the mechanism of free radical scavenging of compounds [9,10,11]. Due to the complex structures of PT, the use of DFT calculation to analyze its characteristic structural units and explore the antioxidant activity and the reaction mechanisms by which antioxidants can exert their activity.

**Figure 1:**
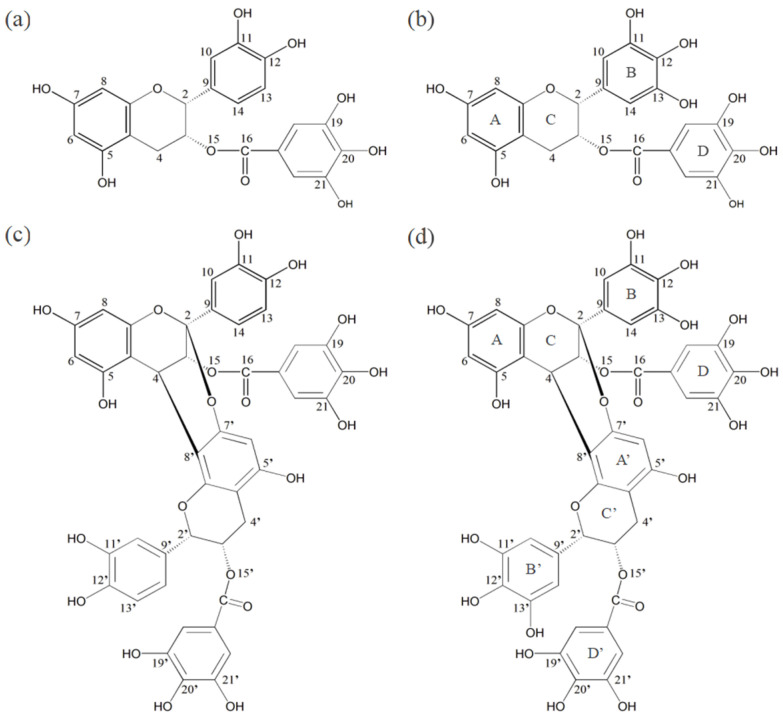
The molecular structure diagrams of the four characteristic structural units from PT: (**a**) ECG, (**b**) EGCG, (**c**) A−ECG dimer, (**d**) A−EGCG dimer.

It has been reported that the PT contains a large number of epicatechin gallate (ECG) and epigallocatechin gallate (EGCG) monomers, the corresponding structures of the A−type linked ECG dimer (A−ECG dimer) and A−type linked EGCG dimer (A−EGCG dimer) are very unique [11] and they can be precisely separated in the experiment [6,12]. The molecular structures of the four characteristic structural units are shown in Figure 1. Wang et al. [13] predicted the free radical scavenging capacity of catechins and their derivatives from their structures based on geometries, molecular electrostatic potential (MEP), bond dissociation enthalpy (EDE), the highest occupied molecular orbital (HOMO) and lowest unoccupied molecular orbital (LUMO) and reactivity indices by DFT studies, and found that the B ring of catechins is a stronger electron donor than the A or D ring. Boulmokh et al. [14] combined DFT calculations with antioxidant experiments and showed that EGCG is a more potent antioxidant than epicatechin (EC) and resveratrol. The main active sites of EGCG were gallate moiety and the C4′−OH, while the main active sites of EC were the two phenolic hydroxyl groups of the B ring. Although DFT has been applied to the study of the antioxidant activity of different flavonoids [15,16,17], theoretical studies on the antioxidant capacity of the four characteristic structural units from PT are still quite scarcely reported.

In this study, the molecular structures of the four characteristic structural units from PT (ECG monomer, EGCG monomer, A−ECG dimer, and A−EGCG dimer) and their respective dehydrogenated radicals were optimized by using a DFT approach at the level of the M06−2X/6−311G(d,p) motif [18]. Based on the hydrogen atom transfer mechanism, the quantum chemical parameters such as geometric configuration, the HOMO and LUMO, MEP, natural orbitals, and dissociation energy were investigated, we further compared the antioxidant activity and determined the main active sites of four characteristic structural units.

## 2. Materials and Methods

### 2.1. Computational Methods

The structural models of ECG, EGCG, A−ECG dimer, and A−EGCG dimer were constructed by software Gaussview 5.08. The M06−2X method and the 6−311G(d,p) basis set were used for geometric optimization. All geometric structures in the paper were calculated by frequency without imaginary frequencies. All calculations were completed on the Gaussian09 program [19]. The Gaussview5.0 software was used to plot the molecular electrostatic potential and the frontline molecular orbitals.

### 2.2. Computational Models

**Figure 2:**
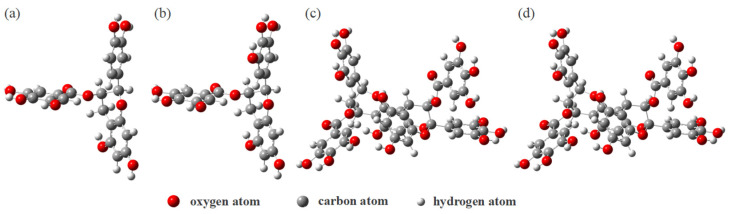
The optimized geometric models of the four characteristic structural units from PT: (**a**) ECG, (**b**) EGCG, (**c**) A−ECG dimer, (**d**) A−EGCG dimer.

ECG and ECGC monomers can be roughly divided into four rings: A, B, C, and D. The difference between the two monomers is that EGCG has an additional phenolic hydroxyl group at the C_13_ position of the B ring. The model of A−ECG dimer refers to the research of Zhang et al. [20]. It is formed by the polycondensation of two ECG monomers. The linking method is to polymerize the C ring of one ECG monomer with the C_7′_ phenolic hydroxyl group on the A ring of another ECG monomer, and a new six-membered ring is formed. The A−EGCG dimer connection is consistent with the A−ECG dimer. The optimized geometric models of ECG, EGCG, A−ECG dimer, and A−EGCG dimer are shown in Figure 2.

## 3. Results and Discussion

### 3.1. Structural Parameters of Molecules

Most of the polyphenols capture radicals by hydrogen atom transfer mechanism, i.e., the phenolic hydroxyl group in the main site loses a positively charged hydrogen atom and is transferred to the oxidative radical [14]. The valence bond theory shows that the longer the O−H bond length of the phenolic hydroxyl group, the smaller the bond energy it contains. That is, the longer the phenolic hydroxyl bond length is, the easier it is for oxygen radicals to extract the hydrogen atom at that position, and the easier it is for antioxidant reactions to occur. Therefore, the analysis of the structural parameters of compounds, especially the bond lengths of phenolic hydroxyl groups at various positions, is one of the effective methods to determine the antioxidant activity of polyphenolic substances. The main structural parameters of the four characteristic structural units from PT by structural optimization are listed in Table 1.

As shown in Table 1, the bond lengths of some phenolic hydroxyl groups on the B and D rings of ECG and EGCG are relatively long, so the B and D rings of these two monomers are more likely to undergo antioxidant reactions. From the dihedral angle D_4-3-2-9_ data and geometric configuration diagrams, it can be seen that the D rings (gallic acyl functional group) of both monomers have an angle with the A and C rings, with a maximum angle of 81°. The acyl group of the D ring tends to form intramolecular hydrogen bonds with the adjacent phenolic hydroxyl group, resulting in the difficult removal of the hydrogen atoms of the related phenolic hydroxyl group. Therefore, the phenolic hydroxyl group of the B ring in ECG and EGCG is more likely to undergo a hydrogen abstraction reaction. The analysis of the EGCG geometric structure shows that the hydrogen of the C_12_ phenolic hydroxyl group is prone to form intramolecular hydrogen bonds with the neighboring C_11_ phenolic hydroxyl group, which leads to the bond length of the C_12_ phenolic hydroxyl group being extended and its reactivity being reduced. Even though R_C12_−OH > R_C11_−OH, the C_11_ phenolic hydroxyl group is the most reactive among the ECG monomers. The same is true for the EGCG monomer, where the bond length of the phenolic hydroxyl group at the C_13_ position of the B ring is longer than that of the C_12_ position. However, the hydrogen of the C_13_ phenolic hydroxyl group is hardly captured due to the formation of intramolecular hydrogen bonds, and thus the C_12_ phenolic hydroxyl group is more active. 

In addition, the longest bond lengths of phenolic hydroxyl groups on both ECG dimer and EGCG dimer are located at the C_19′_ position of the D’ ring. Due to the presence of intramolecular hydrogen bonds among three phenolic hydroxyl groups on the D’ ring of the dimer, the C_20′_−position phenolic hydroxyl group whose bond length is slightly smaller than C_19′_ is more active. In contrast, the bond length of the C_5_ phenolic hydroxyl group of the dimer A ring is only slightly smaller than that of C_19′_, so the C_5_ phenolic hydroxyl group may also be the reactive site of both dimers. In view of the above phenolic hydroxyl bond lengths of the main active sites, the activity magnitudes of the four characteristic structural units are: A−ECG dimer > A−EGCG dimer > ECG > EGCG.

### 3.2. Frontier Molecular Orbitals

Frontier molecular orbitals analysis using Gaussview5.0 software is an effective method for understanding intramolecular reactivity and electron hopping. Frontier molecular orbitals (HOMO and LUMO) are the most important parameters of the molecular electronic structure, and their energy gap describes the reactivity of the compound. The higher the HOMO energy of a molecule, the easier it loses electrons and the faster the reaction of electron-donating is. On the other hand, the lower LUMO energy of a molecule indicates its ability to accept electrons [21,22]. The amount of energy required to jump from HOMO to LUMO can be determined by the magnitude of the energy level difference Δ*E*_(LUMO−HOMO)_ between the two. The high reactivity of compounds is characterized by a small energy gap (Δ*E*_(LUMO−HOMO)_ = Δ*E*_HOMO_ − Δ*E*_LUMO_) between the HOMO and the LUMO energies, this means that these compounds can behave as soft electrophiles [14]. The detailed HOMO and LUMO energies of the four characteristic structural units of persimmon tannins and their gaps are shown in Table 2.

Comparing the Δ*E*_(LUMO−HOMO)_ energies of the compounds, it is somewhat possible to infer the reactivity of the compounds. As can be seen from Table 2, the values of the Δ*E*_(LUMO−HOMO)_ energies of the four characteristic structural units are ranked: Δ*E*_(LUMO−HOMO)_ (A−ECG dimer) < Δ*E*_(HOMO)_ (A−EGCG dimer) < Δ*E*_(HOMO)_ (ECG) < Δ*E*_(HOMO)_ (EGCG). The Δ*E*_(LUMO−HOMO)_ values of two A−linked dimers are significantly smaller than that of the two monomers which indicate that the dimers are more reactive. Meanwhile, the results reflect the same antioxidant activity as that analyzed in the geometric configuration. In addition, the strength of the antioxidant activity of the two monomers is also consistent with the study of Wang et al. [13].

**Figure 3:**
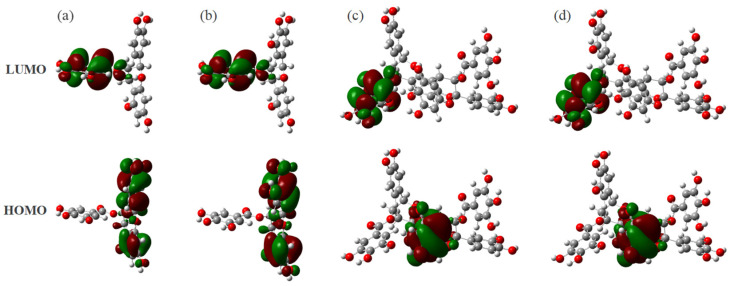
The frontier molecular orbital structure of the four characteristic structural units from PT: (**a**) ECG, (**b**) EGCG, (**c**) A−ECG dimer, (**d**) A−EGCG dimer.

In addition, it was reported that antioxidant activity of flavonoids is mainly related to the presence of hydroxyl groups [14,23], there are many phenolic hydroxyl groups on the structures of the two dimers and some phenolic hydroxyl hydrogen atoms have more positive charges, we analyze the frontier molecular orbitals of those compounds to determine whether these phenolic hydroxyl groups have the opportunity to become the main reaction sites. As shown in Figure 3a,b, the HOMO and LUMO of both monomers are mainly concentrated in the A, B, and D rings, so the phenolic hydroxyl groups on these three rings are active. In contrast, as shown in Figure 3c,d, the two dimers have large structural changes after polymerization, so their frontier molecular orbital structure diagrams are different from those of the monomers. The HOMO and LUMO of the dimer are mainly concentrated in the two rings A’ and D’. The other positions without distributed electron clouds indicate that the phenolic hydroxyl groups at these positions are inactive, and therefore these sites are not considered when performing the analysis.

### 3.3. Molecular Electrostatic Potential (MEP)

The MEP diagrams of four characteristic structural units from PT are plotted by Gaussview5.0 software, which can effectively and visually explore the reactivity of those compounds. The red color indicates electron-rich sites, and the blue color indicates electron-deficient sites. Therefore, the darker the blue position is, the more positive charges it carries, and the more likely it is to be attacked by negatively charged groups (oxygen free radicals), while the deeper the red position is, the more likely the nucleophilic reaction will occur [24].

**Figure 4:**
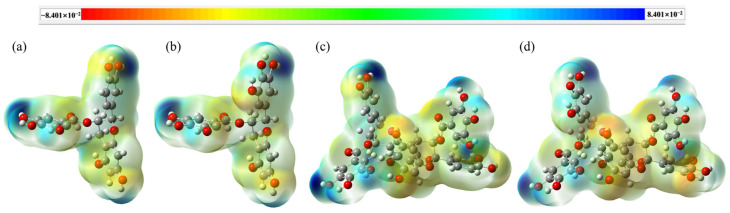
The MEP diagrams of the four characteristic structural units from PT: (**a**) ECG, (**b**) EGCG, (**c**) A−ECG dimer, (**d**) A−EGCG dimer.

Figure 4a,b shows that the blue color (more positively charged) is concentrated on the phenolic hydroxyl groups of the B and D rings, so the main active sites of ECG and EGCG are located here. Figure 4c,d shows that the main active positions of the two dimers are on the phenolic hydroxyl groups of the A, B’, and D’ rings. However, the aforementioned frontline molecular orbital analysis reveals that the HOMO and LUMO electron clouds do not exist on the B’ rings of these two dimers, indicating that the phenolic hydroxyl groups on the B’ ring are not the main active site for hydrogen capture by oxygen radicals, so it is not considered in the subsequent analysis.

### 3.4. Natural Bond Orbital (NBO) of Phenolic Hydroxyl Hydrogen Atoms

It is well known that oxygen radicals account for the majority of free radicals in the human body. Because the oxygen atoms are negatively charged, the oxygen radicals are very likely to react with the positively charged phenolic hydroxyl hydrogen atoms, which corresponds to the hydrogen atom transfer mechanism between radicals and phenolic hydroxyl groups. Generally speaking, the more positively charged are carried by the phenolic hydroxyl hydrogen atom of the characteristic structural unit, the easier it is for the oxygen radical to react with it, and this position is the main active position of the characteristic structural unit [25]. The observation of the MEP can only give a rough idea of the active sites of the compounds and cannot give an exact value. Therefore, it becomes necessary to analyze the NBO of the phenolic hydroxyl atoms of these four characteristic structural units. The NBO charge numbers of some of the phenolic hydroxyl hydrogen atoms on the four characteristic structural units are given in Table 3. 

From Table 3, it can be seen that the NBO charge number of the D ring phenolic hydroxyl hydrogen atom of ECG monomer is larger than that of the B ring, but considering the existence of the conjugated system in the configuration, the B ring phenolic hydroxyl of both monomers is more likely to react. The NBO charge number of the C_11_ phenolic hydroxyl hydrogen atom of the B ring (0.47874 e) is slightly lower than that of the C_12_ position, and the C_12_ phenolic hydroxyl is more likely to react with free radicals by simply NBO charge analysis. However, there is an intramolecular hydrogen bond between the C_11_ phenolic hydroxyl group and the hydrogen atom of the C_12_ phenolic hydroxyl group. Even though the C_12_ phenolic hydroxyl group has more positive charges, its reactivity is still smaller than that of the C_11_ position, so the C_11_ phenolic hydroxyl group is the main active site of the ECG monomer. In the EGCG monomer, although there are also intramolecular hydrogen bonds between the ophthalmic trials of the B ring, the NBO charge on the hydrogen atom of the C_12_ phenolic hydroxyl group is much larger than that of the C_11_ and C_13_ positions, so it can be judged that the main active site of EGCG is located at the C_12_ phenolic hydroxyl group, which is consistent with the previous analysis. In addition, both A−linked dimers also have an o-phenylene triol on the D’ ring, while the NBO charge on the hydrogen atom of the C_20′_ phenolic hydroxyl group is greater than that of the other phenolic hydroxyl groups, so we judge that the main active site of both dimers is the phenolic hydroxyl group at the C_20′_ position. The order of the number of positive charges on the active site of these four compounds is A−ECG dimer > A−EGCG dimer > EGCG > ECG. Since the charge number of the C_11_ phenolic hydroxyl hydrogen atoms on the ECG monomer is smaller than that of the C_12_ phenolic hydroxyl group on the EGCG, resulting in a smaller antioxidant activity of ECG than EGCG. This is slightly different from the results of structural parameter analysis, so we need to conduct subsequent analysis to make a judgment.

### 3.5. The Bond Dissociation Energy of Phenolic Hydroxyl Groups

To evaluate the bond energy of a phenolic hydroxyl group, in addition to the bond length of the O−H bond, another factor is its bond dissociation energy (BDE). The magnitude of the BDE reflects the ease of hydrogen abstraction of the phenolic hydroxyl group from the oxygen radical and the stability of the radical of the compound after dehydrogenation. In general, the lower the BDE of different phenolic hydroxyl groups of the same substance is, the easier the hydrogen atom of the phenolic hydroxyl group at that position can be seized, and the stronger its antioxidant activity is [13]. The dissociation energy is calculated as follows:

(1) $$\mathrm{BDE}=E_{sr}+E_{Hr}-E_{s}$$

where *E_sr_*, *E_Hr_*, and *E_s_* represent the energies of the radicals of the compounds after dehydrogenation, the energies of the hydrogen atoms and the energies of the four compounds, respectively. In this paper, the M06−2X/6−311G(d,p) method was used to optimize the calculation of the radicals of the four compounds after dehydrogenation of phenolic hydroxyl groups at different positions, and zero-point vibrational energy correction was performed. The BDE values of the relevant phenolic hydroxyl groups are shown in Table 4.

From the data in Table 4, it can be seen that BDE(C_11_−OH) is the smallest among ECG monomers with only 0.134529 a.u. Therefore, the reactivity of the phenolic hydroxyl group at the C_11_ position is the strongest among ECG. Analysis of the BDE of the other three compounds reveals that the main active site of the EGCG monomer is located at the C_12_ phenolic hydroxyl, while the main active sites of both dimers are located at the C_20′_ position of the D’ ring phenolic hydroxyl, which was consistent with the main reactive sites analyzed previously. When comparing the two monomers, the minimum dissociation energy of ECG (0.134529 a.u.) is smaller than that of EGCG (0.137218 a.u.), so the reactivity of ECG is larger than that of EGCG. Similarly, when comparing the two A−linked dimers, the minimum dissociation energy of the ECG dimer is smaller than that of the EGCG dimer, so the reactivity of the ECG dimer is larger. However, when the monomer and dimer are compared together, the minimum dissociation energy of the dimer is larger than that of the monomer, because the polymerization of the dimer leads to a larger change in the molecular structure, and the polymerization also increases the spatial resistance, so this comparison is not meaningful.

## 4. Conclusions

In this work, the antioxidant activities of EGCG, ECG, A−ECG dimer, and A−EGCG dimer from PT were investigated by calculating and analyzing their geometric parameters, frontier molecular orbitals, MEP, NBO, and BDE by using the DFT method. The geometric parameters of the structural units, MEP, and the frontier molecular orbitals show that the preferential active sites of the ECG and EGCG monomers are the B ring in the phenolic hydroxyl groups, and the main active sites of the two dimers were the A and D’ rings on the phenolic hydroxyl groups. The NBO and BDE calculations of phenolic hydroxyl hydrogen atoms show that the C_11_ phenolic hydroxyl group on ECG monomer and C_12_ phenolic hydroxyl group on EGCG monomer are more favored sites, while the main active site of both A−linked dimers is the D’ ring C_20′_ phenolic hydroxyl group. Based on the antioxidation mechanism of hydrogen atom transfer and the quantum chemical parameters, the antioxidant potential of the four characteristic structural units from PT shows the A−ECG dimer has the highest antioxidant capacity.

## Figures and Tables

**Table 1:** The main structural parameters of the four PT characteristic structural units.

	ECG	EGCG	A−ECG Dimer	A−ECG Dimer
R_C5_−OH	0.96022	0.96021	0.97281	0.97256
R_C7_−OH	0.96048	0.96046	0.97196	0.97205
R_C11_−OH	0.95988	0.95996	0.96676	0.96682
R_C12_−OH	0.96433	0.96349	0.97068	0.97030
R_C13_−OH	−	0.96423	−	0.97080
R_C19_−OH	0.96411	0.96412	0.97085	0.97074
R_C20_−OH	0.96427	0.96421	0.97089	0.97083
R_C21_−OH	0.96040	0.96036	0.96704	0.96699
R_C19′_−OH	−	−	0.97324	0.97314
R_C20′_−OH	−	−	0.97128	0.97121
R_C21′_−OH	−	−	0.96732	0.96726
D_4-3-2-9_	177.05255	176.23593	−176.13482	−176.25620
D_4-3-15-16_	−81.30450	−80.94205	−78.78486	−78.95503
D_1-2-7′-5′_	−	−	−86.92627	−86.80530

**Table 2:** The highest occupied orbital energy, the lowest empty orbital energy, and the difference between the two (a.u.).

	ECG	EGCG	A−ECG Dimer	A−EGCG Dimer
Δ*E*_(LUMO)_	−0.00967	−0.00834	−0.01853	−0.01882
Δ*E*_(HOMO)_	−0.26608	−0.26621	−0.24692	−0.24728
Δ*E*_(LUMO−HOMO)_	0.25641	0.25787	0.22839	0.22846

**Table 3:** The NBO charge on the hydrogen atoms of phenolic hydroxyl groups (e).

	ECG	EGCG	A−ECG Dimer	A−EGCG Dimer
H atom of C_5_−OH	0.47085	0.47075	0.50132	0.50142
H atom of C_7_−OH	0.46627	0.47075	0.48776	0.48819
H atom of C_11_−OH	0.47974	0.47945	0.48034	0.48096
H atom of C_12_−OH	0.48122	0.49287	0.48326	0.49387
H atom of C_13_−OH	−	0.48211	−	0.48249
H atom of C_19_−OH	0.48326	0.48274	0.48361	0.48279
H atom of C_20_−OH	0.49639	0.49591	0.49704	0.49448
H atom of C_21_−OH	0.48357	0.48314	0.48400	0.48346
H atom of C_19′_−OH	−	−	0.49534	0.49505
H atom of C_20′_−OH	−	−	0.50472	0.50315
H atom of C_21′_−OH	−	−	0.48661	0.48616

**Table 4:** The BDE of phenolic hydroxyl groups at different positions of four persimmon tannin characteristic structural units (a.u.).

	ECG	EGCG	A−ECG Dimer	A−EGCG Dimer
BDE(C_5_−OH)	0.147299	0.147108	0.154702	0.154792
BDE(C_7_−OH)	0.154142	0.154006	0.154463	0.154576
BDE(C_11_−OH)	0.134529	0.137308	−	−
BDE(C_12_−OH)	0.150097	0.137218	−	−
BDE(C_13_−OH)	−	0.15258	−	−
BDE(C_19_−OH)	0.153795	0.154378	−	−
BDE(C_20_−OH)	0.140509	0.140191	−	−
BDE(C_21_−OH)	0.142142	0.141735	−	−
BDE(C_19′_−OH)	−	−	0.149704	0.149647
BDE(C_20′_−OH)	−	−	0.142017	0.142053
BDE(C_21′_−OH)	−	−	0.143425	0.143471

## Data Availability

The data presented in this study are available upon request from the corresponding author.
